# Chronic health conditions and health-related economic inactivity in midlife: Evidence from the 1958 and 1970 British birth cohorts

**DOI:** 10.1016/j.ssmph.2026.101940

**Published:** 2026-06-23

**Authors:** Laura Gimeno, Charis Bridger Staatz, Alice Goisis, Jennifer B. Dowd, George B. Ploubidis

**Affiliations:** a Centre for Longitudinal Studies, Social Research Institute, University College London, 55-59 Gordon Square, London, WC1H 0NU, UK; b Leverhulme Centre for Demographic Science, Nuffield Department of Population Health, University of Oxford, 42-43 Park End Street, Oxford, OX1 1JD, UK

**Keywords:** Economic inactivity, Mental health, Chronic disease, Longitudinal data, Birth cohort

## Abstract

**Background:**

Rising health-related economic inactivity (IN-HLT) is a growing concern in the United Kingdom, yet little is known about whether its relationship with chronic health conditions has changed across generations.

**Methods:**

Using data from two large birth cohort studies representative of people born in Britain in 1958 (*n* = 9761) and 1970 (*n =* 7336), we quantified associations between physical and mental ill-health at age 42 with IN-HLT at age 50-54 using multinomial logistic regression models, adjusting for previous economic activity and a rich set of lifecourse sociodemographic characteristics, and estimated average marginal effects (AME).

**Results:**

Ill-health, particularly longstanding illness and mental ill-health, were associated with a higher risk of IN-HLT in both cohorts. Having a longstanding illness at age 42 was associated with 5 to 6 percentage-point higher risk of IN-HLT (AME_1958_ = 5.1 [95% Confidence Interval (CI) 2.1, 8.1], AME_1970_ = 6.5 [95%CI 3.4, 9.6]), and psychological distress with a 4 percentage-point higher risk (AME_1958_ = 3.8 [95%CI 0.4, 7.1], AME_1970_ = 4.5 [95%CI 1.1, 7.8]). Except for high blood pressure, which was associated with IN-HLT in the 1958 cohort only, the magnitude of associations between chronic conditions and IN-HLT was similar across generations despite them experiencing different economic and policy contexts.

**Implications:**

Given increases in obesity and mental ill-health prevalence across successive British birth cohorts, our findings suggest that both primary prevention and policies to better support individuals with chronic health conditions to remain and return to economic activity are needed to sustain later-life labour force participation.

## Highlights

• Economic inactivity due to health problems (IN-HLT) has increased in the UK.
• We quantify ill-health's (age 42) impact on IN-HLT (age 50-54) across British cohorts born in 1958 and 1970.
• Chronic physical and mental health conditions increased IN-HLT risk in both cohorts.
• Except for high blood pressure, association magnitudes were similar across cohorts.
• This is despite higher prevalence of ill-health in the younger cohort.

## 1 Background

Rising health-related economic activity (IN-HLT) and benefit expenditure are a growing policy concern in the United Kingdom (UK). In February-April 2024, 2.83 million (6.6%) people aged 16-64 were not working due to long-term health problems including mental ill-health, cardiovascular disease and musculoskeletal problems, up from 2 million in Spring 2019; ONS, 2022, 2023, 2024), with the largest increase in absolute terms in the 50-64 age-group (OBR, 2023). The age-specific proportion of adults claiming health-related benefits has increased (Ray-Chaudhuri & Waters, 2024), as has health-related benefit expenditure (OBR, 2023). While many individuals who are IN-HLT receive health-related benefits, this overlap is not complete, with 1-in-3 not receiving disability benefit, and 1-in-5 not receiving incapacity benefit (OBR, 2023). IN-HLT is a policy-relevant outcome, since it excludes those who work, even part-time, and captures individuals who are not working due to their health, but who may be assumed to be inactive for other reasons if not in receipt of health-related benefits.

Rising IN-HLT presents an important fiscal challenge since it is linked to lower revenue from tax and National Insurance contributions, compounding the effects of population ageing on the size of the labour force, and increased benefit expenditure. In July 2023, the UK Office for Budget Responsibility estimated that “the increase in working-age inactivity due to long-term sickness since the pandemic (alongside rising ill-health among those in work) [had] already added £6.8bn to the annual welfare spend, cost £8.9bn in foregone tax receipts, and therefore added £15.7bn (0.6% of GDP) to annual borrowing” (OBR, 2023). While IN-HLT can be necessary and inevitable, reducing the number of people who are IN-HLT – particularly at older ages – by improving population health and by better supporting people with chronic conditions to stay in work, constitutes a potentially attractive opportunity to mitigate the impact of population ageing on labour supply (André et al., 2024).

The extent to which increases in IN-HLT and benefits claims in the UK are driven by long-term worsening population health is an area of active debate (Atwell et al., 2023; Cribb, 2023). Obesity, mental ill-health, and multimorbidity prevalence have increased across cohorts (Johnson et al., 2015; McElroy et al., 2023; Ribe et al., 2024) and previous increases in healthy life expectancy and life expectancy free from work-limiting conditions have stalled (Lynch et al., 2022; Welsh et al., 2021), indicative of a growing working-age population at risk of IN-HLT.

Another mechanism to consider are possible changing associations between ill-health and IN-HLT. For instance, the likelihood of IN-HLT among those with chronic conditions may have increased over time due to increasing severity or complexity of health problems, or other social mechanisms (e.g., limited support to remain in work, discrimination). On the contrary, policy changes coupled with a shift toward non-manual occupations in more recently born cohorts could mean that those with chronic conditions are less likely to be IN-HLT than they might have been in the past. Since health-related benefits were first introduced in the UK in 1971, policy changes have tended to increase the conditionality and decrease the generosity of these benefits, and legislated against discrimination on the grounds of disability (Fig. S1).

It is well-established that individuals with health problems are more likely to be economically inactive, to have less stable employment trajectories (Gondek et al., 2018), and to drop out of the labour market for a variety of reasons, including early retirement (e.g., Blundell et al., 2023; Jones et al., 2020; Rice et al., 2011; Stafford et al., 2017; Trevisan & Zantomio, 2016). There is comparatively little evidence on how the relationship between chronic health conditions and economic activity, particularly IN-HLT, has changed over time, despite the policy-relevance of this question. Indeed, given increases in the prevalence of chronic health problems among working-age individuals, weakening the ties between ill-health and IN-HLT is crucial to supporting labour market participation. Existing studies have used repeated cross-sectional data (Minton et al., 2012), such that the impact of economic activity on the reporting of ill-health cannot be ruled out, or focused on adolescent mental ill-health rather than midlife chronic health conditions (Pierce et al., 2025; Thompson et al., 2023).

We aimed to fill this research gap using prospectively collected data from two birth cohort studies representative of people born in Britain in 1958 and 1970 to quantify associations between chronic health conditions at age 42 on IN-HLT at age 50-54, more than a decade before State Pension Age, in two generations. We hypothesised that associations would be weaker in the later-born cohort, due to improvements in the diagnosis and treatment of chronic health conditions, changes in the types of occupation of cohort members, and policy changes promoting work participation and dissuading labour market drop out.

## 2 Data and methods

### 2.1 Data

We used data from the 1958 National Child Development Study (1958c) and the 1970 British Cohort Study (1970c), which have followed all people born in England, Wales and Scotland in one week of 1958 (*n* = 18,558) and 1970 (*n =* 18,037) from birth. These studies have been described in detail elsewhere (Elliott & Shepherd, 2006; Power & Elliott, 2006; Sullivan et al., 2023). We used data collected up to age 50-51 (2008/9) in the 1958c, and 51-54 (2021/4) in the 1970c. Whilst age overlap was not exact, these data collection sweeps correspond to the first surveys in cohort members’ fifties in each study.

**Fig. 1:**
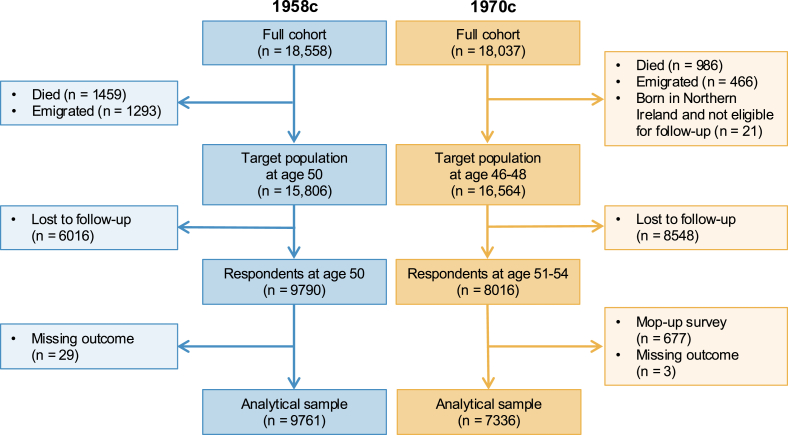
Derivation of the analytical sample in the 1958c and 1970c.

Το describe the prevalence of poor health and the distribution of economic activity at age 42, we used data from all respondents at age 42 in the 1958c (*n =* 11,419) and 1970c (*n =* 9841). Analyses quantifying associations between health at age 42 and subsequent economic activity used data from all respondents at ages 50-54 (*n*_*1958c*_ = 9761; *n*_*1970c*_ = 7336) with information on current economic activity (Fig. 1).

### 2.2 Outcome

The outcome was self-reported economic activity at age 50-54, grouped into.

• Active full-time (ACT-FT): employed or self-employed working >30 h/week.
• Active part-time (ACT-PT): employed or self-employed working ≤30 h/week.
• Unemployed and looking for work (UNEMP).
• Inactive due to health (IN-HLT): economically inactive due to long-term or short-term sickness.
• Inactive due to other reasons (IN-OTH): economically inactive due to full-time education, training schemes, looking after home or family, retired, or other.

### 2.3 Exposures

Our analyses focused on self-reported health conditions which are typically cited as reasons for IN-HLT in cross-sectional data: mental ill-health, cardiometabolic disease, and musculoskeletal disease (ONS, 2023).

• ***Longstanding illness*.** Reported any longstanding illness or disability at age 42.
• ***Psychological distress****.* Score ≥4 on the 9-item Malaise Inventory (Rutter et al., 1970), measuring non-specific symptoms of depression, anxiety and distress. Examinations of measurement equivalence suggest that members of the 1958c and 1970c interpret questionnaire items similarly (Ploubidis et al., 2019).
• ***Obesity*.** Body mass index ≥30 kg/m^2^ at age 42.
• ***Diabetes****.* Ever had diabetes up to and including age 42.
• ***High blood pressure****.* Ever had high blood pressure up to and including age 42.
• ***Back pain****.* Ever had persistent back pain, sciatica, or slipped disc up to and including age 42.

More information on the exposures can be found in Table S1. It is worth noting that these exposure groups also included those with comorbidities, and that the comparator group was not “healthy” since it included all individuals without the chronic condition of interest, including those with other health conditions.

### 2.4 Covariates

A range of socioeconomic and demographic variables associated with both health status and economic activity were included in the analysis (Table 1). Since the 1958c and 1970c were designed to be representative of births occurring in Britain in those years and did not oversample racial/ethnic minority participants, cohort members are predominantly white (>95%; Elliott & Shepherd, 2006; Power & Elliott, 2006; Sullivan et al., 2023), so we did not control for race/ethnicity in our analyses.

**Table 1:** Covariates in fully-adjusted regression models.

Variable	Definition	Age measured	
		1958c	1970c
Economic activity (lagged outcome)	ACT-FT, ACT-PT, UNEMP, IN-HLT, IN-OTH	42	42
Sex at birth	Male, female	0	0
Father's social class	Professional/managerial/intermediate/skilled (RGSC I-III), partly skilled/unskilled (RGSC IV-V)	0	0
Cognitive ability	Principal component 1 from PCA of cognitive test scores	11	10
Region of residence	North/North West/Yorkshire and Humberside, West Midlands/South West, East Midlands/East Anglia, South East/London, Wales/Scotland/Northern Ireland	42	42
Partnership status	Married/cohabiting, other	42	42
Educational attainment	NVQ levels 0-3, NVQ level ≥4	42	42
Equivalised household income	Quintile of equivalised household income. Includes income from cohort member, their partner, and any additional sources of income (e.g., benefits), and is related back to the size of the household.	42	42
Housing tenure	Owns home or is buying home with the help of a mortgage, other.	42	42
Children in household	Whether there are any (biological or non-biological) children resident in the household.	42	42
Occupation	Non-manual (RGSC I-IIINM), non-manual (IIIM-V)	42	42
Age at outcome measurement	Age when the outcome was measured in years (year surveyed minus year of birth) for BCS70 only	NA	51-54

**Note:** ACTFT = active full-time. ACTPT = active part-time. UNEMP = unemployed. INHLT = inactive for health reasons. INOTH = inactive for other reasons. RGSC = Registrar General Social Class 1990. PCA = Principal Component Analysis. NVQ = National Vocational Qualification.

### 2.5 Descriptive analysis

We described the prevalence of poor health at age 42, the distribution of economic activity at ages 42 and 50-54 in in both cohorts, the prevalence of poor health at age 42 within economic activity categories at age 50-54, and the distribution of cohort members with chronic health conditions at age 42 across economic activity categories at age 50-54. We quantified the proportion of cohort members who were inactive due to health problems at age 50-54 who were already in this category at age 42.

### 2.6 Main analysis

In each cohort, we examined how health status at age 42 was associated with economic activity a decade later using multinomial regression models. We focus on IN-HLT as the primary outcome of interest due to its current policy relevance but briefly report results for other outcome categories.

**Fig. 2:**
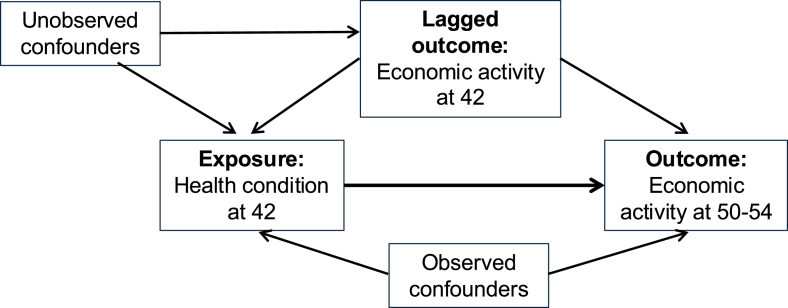
Directed Acyclic Graph showing proposed relationship between variables. **Note:** Observed confounders are the demographic, health and socioeconomic variables shown in Table 1.

For each exposure in turn, we constructed unadjusted models including only economic activity at age 50-54 (the outcome, with ACT-FT as the reference category) and binary indicators of chronic health conditions. In fully-adjusted models, we additionally included a lagged outcome (economic activity at age 42), and demographic and socioeconomic confounders from across the lifecourse including early-life factors such as cognitive ability and parental social class, as well as sex at birth (Table 1). The lagged outcome was included to (i) account for the bi-directional relationship between health and economic activity, recognising that individuals with health conditions were more likely to be inactive at baseline and that economic activity could impact how health is reported; and, crucially, (ii) to block pathways from unmeasured confounders to the outcome, as illustrated in Fig. 2, thereby strengthening causal inference. The inclusion of a broad set of life course confounders in the fully adjusted model also enhances the plausibility that the adjusted associations reflect causal effects, by reducing the risk of residual confounding.

We report results as average marginal effects (AMEs) which estimate the percentage-point difference in the risk of IN-HLT between those with and without the exposure. AMEs were obtained using the mimrgns command (Klein, 2014) in Stata version 18.0 (StataCorp, College Station, TX), which calculates standard errors using the delta method and pools results across imputed datasets following Rubin's rules (Rubin, 1987; see Section 2.9 for further information on how missing data was handled).

### 2.7 Secondary analysis

We described the gender and socioeconomic composition of two groups within and across birth cohorts: those with poor health at age 42 in the ACT-FT group at age 50-54, and those with poor health in the IN-HLT group at age 50-54.

### 2.8 Sensitivity analysis

We carried out a series of sensitivity analyses, including (1) replicating analyses when ACT-FT, ACT-PT and UNEMP were grouped into a single economically active category; (2) adjusting for additional potential early-life confounders (maternal and paternal education, housing tenure at age 5/7, breastfeeding, and maternal smoking); (3) restricting the analytical cohort to those who were ACT-FT, ACT-PT or UNEMP at age 42 to remove the impact of reclassifications across economic inactivity categories between ages 42 and 50-54, and (4) excluding those IN-HLT at age 42. We also explored whether the direction of associations was consistent across gender and socioeconomic subgroups for longstanding illness and psychological distress.

### 2.9 Missing data strategy

Like all longitudinal studies, the 1958c and 1970c have experienced loss to follow-up, the likelihood of which varies by individual level characteristics. To mitigate the impact of loss to follow-up, we capitalised on the richness of the cohort data and the known properties of cohort members, using multiple imputation by chained equations (MICE) to handle missing data among sweep respondents, and inverse-probability weights (IPWs) to handle sweep non-response (Katsoulis et al., 2026; Mostafa et al., 2021). More information on the missing data strategy can be found in the Supplementary Material (Text S1, Tables S2–S3).

## 3 Results

### 3.1 Descriptive analysis

**Fig. 3:**
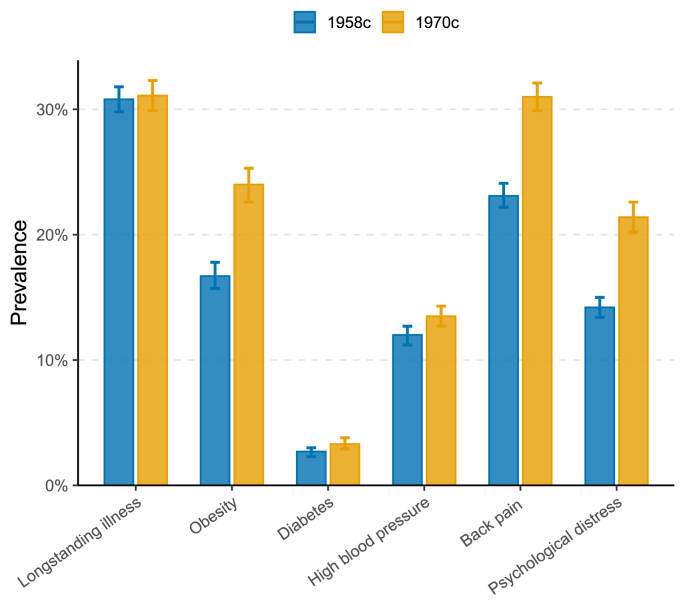
Prevalence of chronic health conditions at age 42 in the 1958c and 1970c. **Note:** LSI = longstanding illness. HIBP = high blood pressure. PD = psychological distress. Based on multiply imputed and weighted data. Point estimates and 95% confidence intervals are shown in Table S4.

The prevalence of chronic conditions at age 42 was higher in the 1970c compared to the 1958c, except longstanding illness (Fig. 3, Table S4). Those in the 1970c were more likely to be experiencing obesity (23.9% vs. 16.8%) or psychological distress (21.4% vs. 14.2%) and to have histories of back pain (31.0% vs. 23.1%), high blood pressure (13.5% vs. 11.9%), and diabetes (3.3% vs. 2.6%, 95% CIs overlap).

**Fig. 4:**
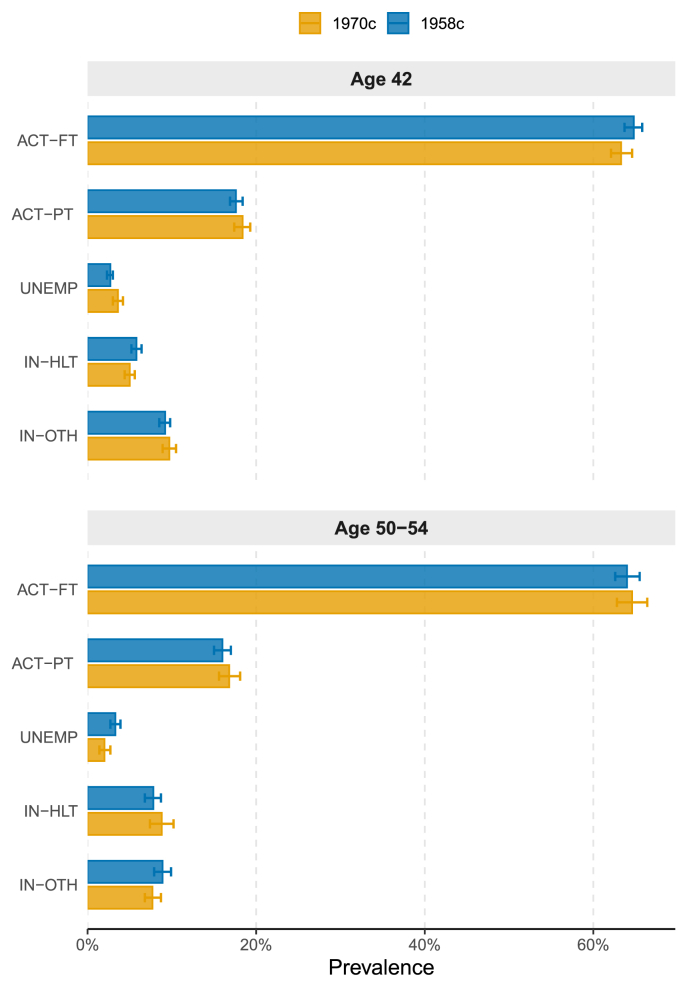
Distribution of economic activity at ages 42 and 50-54 in the 1958c and 1970c. **Note:** ACT-FT = active full-time. ACT-PT = active part-time. UNEMP = unemployed and looking for work. IN-HLT = inactive for health reasons. IN-OTH = inactive for other reasons. Based on multiply imputed and weighted data. Point estimates and 95% confidence intervals are shown in Table S4.

In both cohorts, IN-HLT increased with age (Fig. 4). The distribution of economic activity was similar across cohorts, though the proportion not in work was slightly higher in the 1958c at age 50-51 (in 2008/9) and in the 1970c at age 42 (in 2012), likely due to the 2008 Great Recession. The prevalence of IN-HLT at age 50-54 was similar across cohorts: 7.8% (95% CI 6.8, 8.7) in the 1958c, and 8.8% (95% CI 7.4, 10.2) in the 1970c. In both cohorts, half of those IN-HLT at age 50-54 were already in this same category at age 42 (Table S5).

Among those ACT-FT at age 50-54, the prevalence of self-reported chronic conditions at age 42 tended to be higher in the 1970c (Fig. S2). This partly reflects the general trend of increasing prevalence across cohorts but also suggests that those with chronic conditions at age 42 in the 1970c were more likely to be working, since increases in prevalence could have occurred exclusively among the economically inactive. Indeed, the proportion of individuals ACT-FT was slightly higher in the 1970c for several chronic conditions (though 95% CIs overlapped; Fig. S3). There was also some suggestion that individuals with chronic conditions (except high blood pressure) at age 42 were more likely to be IN-HLT in the 1970c than the 1958c at age 50-54 (though these differences were small and 95% CI overlapped), while the proportion UNEMP or IN-OTH tended to be lower in the 1970c (Fig. S2), which could suggest that there was a change across cohorts in the reasons given for inactivity among people with chronic conditions. In several cases, prevalence of ill-health increased more markedly in the IN-HLT group than in the IN-OTH group, which could lend support to this interpretation (Fig. S3). However, these findings are purely descriptive in nature and do not account for the wide range of demographic and socioeconomic factors besides health status that also drive economic outcomes, such as increases in female labour market participation.

### 3.2 Main analysis

**Fig. 5:**
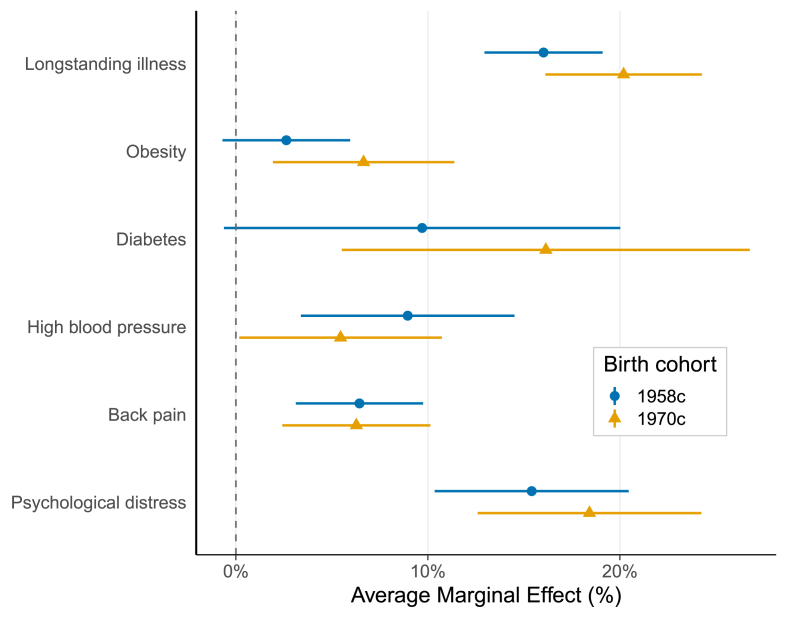
Average marginal effects for chronic health conditions at age 42 and IN-HLT at ages 50-54 from unadjusted models. **Note:** Based on multiply imputed and weighted data. Models include only the exposure and outcome.

In unadjusted models, individuals with chronic conditions were significantly more likely to be IN-HLT in their early fifties in both cohorts compared to those without the condition (Fig. 5). For instance, in the 1970c, having a self-reported longstanding illness at age 42 was associated with a 20 percentage-point higher risk of IN-HLT (AME_1970_ = 20.2 [95% CI 16.1, 24.3]). Having a health condition at age 42 tended to be associated with a lower risk of ACT-FT in both cohorts, and there were no consistent associations with ACT-FT, UNEMP, or IN-OTH. While cohort differences were not formally tested, AMEs for IN-HLT in the 1970c tended to be similar or slightly larger than in the 1958c, except for high blood pressure.

**Fig. 6:**
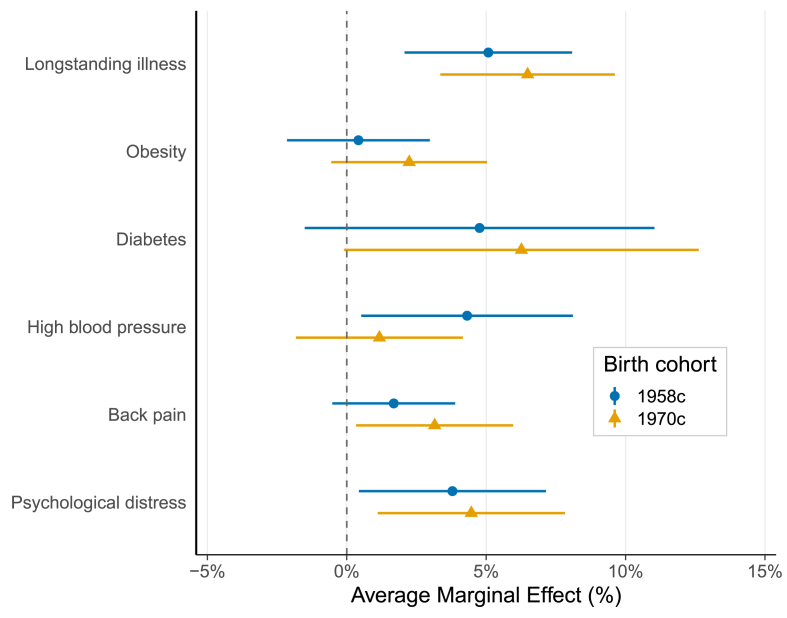
Average marginal effects for chronic health conditions at age 42 and IN-HLT at ages 50-54 from fully adjusted models. **Note:** Models adjust for sex and parental social class, cognitive ability at age 10/11, and education, occupation, household income, housing tenure, partnership status, region of residence, and whether there were any children in the household at age 42. Models for the 1970c also control for age at outcome measurement. Based on multiply imputed and weighted data.

Controlling for sociodemographic characteristics, which differ between cohorts (Table S6), and previous economic activity, strongly attenuated the magnitude of associations between chronic conditions and IN-HLT. Nevertheless, those with chronic conditions at age 42 remained at higher risk of IN-HLT in both cohorts (Fig. 6). For instance, having a longstanding illness at age 42 was associated with a 5 to 6 percentage-point higher risk of IN-HLT a decade later (AME_1958_ = 5.1 [95% CI 2.1, 8.1], AME_1970_ = 6.5 [95%CI 3.4, 9.6]). Back pain (AME_1958_ = 1.7 [95%CI -0.5, 3.9], AME_1970_ = 3.2 [95%CI 0.3, 5.0]) and psychological distress (AME_1958_ = 3.8 [95%CI 0.4, 7.1], AME_1970_ = 4.5 [95%CI 1.1, 7.8]) were also associated with a higher risk of IN-HLT. There was no evidence for an association between obesity and IN-HLT in the 1958c, though there was some suggestion of this in the 1970c (AME_1970_ = 2.2 [95%CI -0.6, 5.0]). While point estimates for diabetes were large (AME_1958_ = 4.8 [95% CI -1.5, 11.0], AME_1970_ = 5.3 [95% CI -0.8, 11.4]), analyses were underpowered and estimates were imprecise due to low prevalence at age 42 in both cohorts. High blood pressure was associated with a higher risk of IN-HLT in the 1958c only (AME_1958_ = 4.3 [95%CI 0.5-8.1]).

As in the unadjusted models, there was some suggestion that having a chronic condition at age 42 was associated with a lower risk of ACT-FT. For instance, those with a longstanding illness at age 42 had a 6 to 7 percentage-point lower risk of ACT-FT at age 50-54 (AME_1958_ = −5.7 [95% CI -9.4, −2.1]; AME_1970_ = −6.9 [95% CI -10.7, −3.2]). No consistent associations with ACT-FT, UNEMP or IN-OTH were observed. While cohort differences were not formally tested, there appeared to be little difference in the magnitude of AMEs between cohorts. If a difference did exist, it suggested that the risk of IN-HLT for those with chronic conditions was higher in the 1970c. The exception to this pattern was high blood pressure.

Risk Ratios for IN-HLT from unadjusted and full-adjusted models are shown in Fig. S4, and results for all outcome categories are shown in the Supplementary Material.

### 3.3 Secondary analysis

Within each cohort, those with chronic conditions tended to be more socioeconomically disadvantaged than the cohort overall and, for psychological distress, were also more likely to be female (Table 2). Compared to all those with chronic conditions, individuals in the ACT-FT group at age 50-54 were more likely to be male and socioeconomically advantaged, while those who in the IN-HLT group were more socioeconomically disadvantaged. Though confidence intervals were large and formal comparisons between cohorts could not be made, there was some suggestion that those with chronic conditions at age 42 were relatively more socioeconomically disadvantaged in the 1970c, particularly for those IN-HLT. The proportion of women in the IN-HLT group was also higher in the 1970c.

**Table 2:** Sociodemographic composition of ACT-FT and IN-HLT groups at age 50-54 by health status at age 42 for selected exposures.

	1958c				1970c			
Health at age 42	Has condition	Has condition	Has condition	Any	Has condition	Has condition	Has condition	Any
Activity at age 50-54	ACT-FT	IN-HLT	Any	Any	ACT-FT	IN-HLT	Any	Any
***Longstanding illness***								
SEX: Male	62.8 (58.8-66.8)	56.4 (48.9-63.9)	50.0 (46.8-53.3)	50.7 (49.2-52.1)	56.8 (52.1-61.5)	44.1 (34.2-54.0)	44.6 (40.8-48.3)	49.6 (47.9-51.3)
EDUCATION: NVQ ≤3	67.9 (64.3-71.4)	91.7 (89.3-94.2)	75.6 (73.4-77.8)	71.4 (70.3-72.5)	56.7 (52.1-61.4)	87.1 (80.5-93.7)	67.5 (64.0-71.0)	63.1 (61.3-64.8)
INCOME: Lowest quintile	17.6 (13.7-21.5)	53.3 (43.6-62.9)	29.6 (26.0-33.2)	21.8 (20.2-23.3)	17.7 (13.3-22.1)	74.2 (65.1-83.4)	36.8 (32.4-41.2)	24.4 (22.2-26.7)
TENURE: Owns home	78.0 (73.4-82.7)	40.6 (29.7-51.6)	67.0 (63.5-70.4)	76.6 (74.8-78.5)	73.8 (68.8-78.7)	18.0 (9.3-26.6)	55.9 (51.6-60.1)	67.1 (64.8-69.4)
OCCUPATION: Manual	38.7 (34.5-42.9)	63.1 (44.8-0.81.3)	41.31 (37.1-45.1)	41.1 (36.0-46.2)	29.7 (25.2-34.3)	55.3 (35.8-74.9)	32.9 (28.3-37.5)	33.6 (30.8-36.4)
***Obesity***								
SEX: Male	64.0 (59.1-68.9)	54.2 (36.7-71.7)	51.5 (46.9-55.2)	50.7 (49.2-52.1)	62.1 (57.1-67.0)	38.7 (23.5-54.0)	50.4 (45.9-54.09)	49.6 (47.9-51.3)
EDUCATION: NVQ ≤3	72.9 (68.5-77.3)	89.1 (83.6-94.6)	78.3 (75.1-81.5)	71.4 (70.3-72.5)	62.9 (58.6-67.2)	90.6 (81.7-99.5)	68.6 (65.0-72.3)	63.1 (61.3-64.8)
INCOME: Lowest quintile	17.0 (12.4-21.5)	47.1 (29.2-65.1)	25.0 (20.7-29.4)	21.8 (20.2-23.3)	17.8 (12.7-22.9)	71.0 (55.5-86.4)	28.9 (24.0-33.8)	24.4 (22.2-26.7)
TENURE: Owns home	78.7 (73.4-83.9)	37.6 (20.9-54.4)	70.3 (64.8-75.7)	76.6 (74.8-78.5)	71.1 (66.0-76.3)	18.0 (6.5-29.6)	61.5 (55.7-66.4)	67.1 (64.8-69.4)
OCCUPATION: Manual	41.4 (35.4-47.4)	60.7 (34.5-87.0)	44.1 (38.9-49.2)	41.1 (36.0-46.2)	37.4 (32.3-42.5)	57.1 (30.3-84.0)	39.1 (33.8-44.4)	33.6 (30.8-36.4)
***Psychological distress***								
SEX: Male	54.6 (47.6-61.5)	51.4 (38.9-63.9)	41.2 (36.1-46.3)	50.7 (49.2-52.1)	56.0 (49.1-62.9)	42.8 (29.2-56.4)	43.8 (38.7-48.8)	49.6 (47.9-51.3)
EDUCATION: NVQ ≤3	73.7 (68.2-79.2)	92.2 (88.9-95.6)	81.1 (78.2-84.0)	71.4 (70.3-72.5)	63.3 (57.8-68.8)	89.1 (81.4-96.8)	72.5 (68.5-76.5)	63.1 (61.3-64.8)
INCOME: Lowest quintile	21.1 (14.4-27.8)	54.1 (40.9-67.4)	35.7 (30.1-41.3)	21.6 (20.2-23.3)	20.3 (13.8-26.8)	79.4 (23.8-35.4)	41.2 (35.6-46.7)	24.4 (22.2-26.7)
TENURE: Owns home	76.0 (68.6-83.4)	29.8 (19.6-40.0)	60.7 (54.8-66.7)	76.6 (74.8-78.5)	68.3 (61.7-75.0)	17.3 (7.7-27.0)	52.8 (47.2-58.3)	67.1 (64.8-69.4)
OCCUPATION: Manual	39.8 (31.8-47.8)	60.2 (35.6-84.8)	43.1 (36.6-49.7)	41.1 (36.0-46.2)	34.9 (28.5-41.4)	59.1 (35.7-82.4)	37.7 (31.7-43.7)	33.6 (30.8-36.4)

**Note:** ACT-FT = active full-time. IN-HLT = inactive for health reasons. Education, household equivalised income, housing tenure, and occupation are measured at age 42. The proportion of cohort members in manual occupations is given among those who were economically active at age 42. Based on multiply imputed and weighted data.

### 3.4 Sensitivity analysis

AMEs for IN-HLT in both cohorts were similar across different model specifications (Fig. S5). In stratified analyses, having a longstanding illness or psychological distress at age 42 was associated with a higher risk of IN-HLT in all subgroups (Fig. S6). Analyses were underpowered to test whether there were differences in associations within strata across cohorts, or within cohorts across strata. Interpretation therefore focuses on the direction of the effect. However, there was some suggestion that the risk of IN-HLT was higher for those who were more socioeconomically disadvantaged, particularly in the 1970c.

## 4 Discussion

### 4.1 Interpretation of the findings

Using data from two birth cohort studies of generations born 12 years apart, we quantified the impact of chronic health conditions at age 42 on risk of IN-HLT a decade later. Cohort members with chronic health conditions were significantly more likely to later be IN-HLT in both cohorts, and there were few differences in the magnitude of associations across generations despite them experiencing different social, policy, economic, and epidemiologic contexts.

The higher prevalence of chronic disease, mental ill-health, and obesity in the 1970c than the 1958c is consistent with a “generational health drift” across post-war generations identified in other studies using the British birth cohorts and other datasets (Gimeno et al., 2024; Gondek et al., 2020; Jivraj et al., 2020).

Descriptive analyses suggested that those with chronic conditions at age 42 in the 1970c were more likely to be ACT-FT in their early fifties. This could be due to a range of factors, such as the changing nature of jobs, provision of reasonable adjustments and declines in health discrimination, better management of chronic conditions themselves, or greater disincentives to leaving the labour market (e.g., increasing conditionality and lower generosity of benefits). However, this also constitutes a greater population at risk of IN-HLT in the 1970c as cohort members grow older, even without accounting for new cases arising between age 42 and 50-54.

Chronic conditions in early midlife were associated with a higher risk of subsequent IN-HLT in both cohorts, even after accounting for previous economic activity and for a rich set of socioeconomic and demographic confounders from across the lifecourse, which increase the plausibility that our findings represent a causal effect of health on IN-HLT under the assumption of no unobserved confounding. This result is consistent with findings from other studies quantifying associations between health and labour market exit in the UK and internationally (Blundell et al., 2023; Jones et al., 2020; van Rijn et al., 2014). Evidence was particularly strong for longstanding illness, and for back pain and mental ill-health, two symptoms-based conditions. Psychological distress was associated with IN-HLT in both cohorts, suggesting that mental ill-health is not only a driver of labour market exit at younger ages, nor has it only emerged as a determinant of IN-HLT in recent years.

We had hypothesised that ill-health would be more weakly associated with IN-HLT in 1970c. However, even after accounting for confounders and previous economic activity, the associations tended to be of similar or greater magnitude in the 1970c compared to the 1958c, except for high blood pressure. This weaker association between high blood pressure and IN-HLT is likely a consequence of improvements in screening, diagnosis, and management, resulting in fewer complications. However, a similar improvement was not seen for other conditions, which may have implications given increasing chronic disease prevalence should this trend of stable disease-inactivity association continue in future cohorts.

We noted that individuals with chronic conditions, particularly those IN-HLT at age 50-54, appeared more socioeconomically disadvantaged in the 1970c. However, changes in the socioeconomic composition of individuals with chronic conditions are unlikely to fully explain our finding, since fully-adjusted models tightly controlled for a wide range of socioeconomic factors in both childhood and adulthood. While few studies have quantified cohort differences in associations between health and socioeconomic outcomes using cohort data, those that have also found no evidence for a weakening association across cohorts. One study quantifying associations between adolescent mental health problems and a range of social and economic outcomes at age 42 in the 1958c and 1970c, similarly found that rather than weaken across cohorts, associations tended to be similar or stronger in the 1970c (Thompson et al., 2023). Another recent study using data from Understanding Society, a large longitudinal study of UK households, found no evidence for a weakening association between mental ill-health and being Not in Employment, Education or Training (NEET) among young people aged 16-24 – between 2010 and 2023, the association appeared to strengthen (Pierce et al., 2025). Associations between adolescent mental health and other important outcomes, including educational attainment and social functioning, have also strengthened over time (Armitage et al., 2025; Sellers et al., 2019).

One possible explanation for the stable association between chronic conditions and IN-HLT across cohorts is that individuals in the 1970c may have more complex health conditions, whether due to the severity of the condition itself or to comorbidities, increasing the risk of complications between age 42 and 50-54, or making it more difficult to manage these conditions whilst in work. Multimorbidity has become more common in more recently born generations (Gondek, 2020; Ribe et al., 2024). Comorbidities are likely an important part of the mechanism linking conditions like hypertension and diabetes to inactivity (Ervasti et al., 2016; Kark et al., 2007), and employment rates are lower among people with multiple health conditions (ONS, 2022). The age of onset of mental ill-health and obesity has also declined across generations, meaning that at the same age, members of the 1970c have been exposed to ill-health for longer on average (Johnson et al., 2015; McElroy et al., 2023), and there is some evidence that, among individuals reporting the same level of health, a previous history of poor health is associated with a greater likelihood of labour market drop out (Disney et al., 2006), highlighting the importance of both current health and health histories.

It is possible that those with chronic health conditions in the 1970c could also perceive their own health as worse, affecting their labour market decisions, however, examinations of measurement equivalence of the Malaise Inventory (from which our measure of psychological distress is derived) suggest that members of the 1958c and 1970c interpret questionnaire items similarly (Ploubidis et al., 2019), making this explanation unlikely in the context of this study.

Another possibility is that associations between chronic conditions and IN-HLT remained similar despite contextual changes due to changes to factors along the causal pathway (e.g., job stress and other factors related to the work environment, timely access to healthcare and social support, knock-on effects of health problems at age 42 on mental wellbeing). Finally, as hinted at in descriptive analyses, it is possible that that there has been a change in how individuals with chronic health conditions report the reason for economic inactivity. For example, someone with chronic health conditions in the 1970c may report being IN-HLT, but the same individual in the 1958c may have stated that they were IN-OTH. While IN-HLT is distinct from benefits receipt, it is likely that the extent to which people self-report being IN-HLT as opposed to IN-OTH is partly influenced by benefit eligibility (OBR, 2023). For instance, the more female composition of the IN-HLT group in the 1970c may partly be a consequence of increasing female labour market participation, which made more women eligible for contributions-based benefits. Though associations between chronic conditions and IN-OTH tended to be non-significant in both cohorts, there was some suggestion that individuals who experiencing obesity or psychological distress at age 42 were less likely to be IN-OTH in the 1970c than in the 1958c.

### 4.2 Strengths and limitations

A major strength of this study is its use of data from two large birth cohorts with similar study designs, but representative of two generations born 12 years apart. We were able to quantify associations between chronic conditions and subsequent economic activity using the same analytical strategy in both datasets, allowing us to explore whether the magnitude of associations might differ across generations. With data collection spanning over fifty years, we were able to examine the impact of health on economic activity nearly a decade later, which is important considering this study's focus on chronic conditions, the effects of which likely play out across long time horizons. The prospective nature and richness of the data meant that the temporal ordering of exposure and outcome could be established, and we could control for a wide range of potential confounders from childhood and adulthood, including cognitive ability, as well as previous economic activity. This strengthens the plausibility of the assumption of no unmeasured confounding – although the possibility can never be ruled out entirely – which is crucial for interpreting the findings as indicative of the causal effect under assumptions of chronic conditions on IN-HLT.

This study also has limitations. The first set of limitations is related to the measurement of chronic conditions. These were self-reported, so could be affected by reporting error. However, awareness of having a health condition (either due to experiencing symptoms or receiving a diagnosis) is likely an important part of the mechanism linking ill-health and labour market outcomes, especially for chronic health conditions that often do not wholly limit the ability to work.

Chronic conditions were operationalised as binary variables and do not differentiate the severity of the condition or between those with or without comorbidities. Our analyses did not account for comorbidities, but cross-sectional analyses of UK data have shown that employment rates are substantially lower among individuals with multiple health conditions (e.g., 29% for those with 5+ conditions compared to 65% for those with just one chronic condition; ONS, 2022). A key avenue for future research should therefore be to consider the impact of multimorbidity on labour market participation, with a special focus on co-occurring physical and mental health conditions (Henderson et al., 2024), which interact in complex and dynamic ways across the life course.

Some retrospective harmonisation was required to enable cohort comparisons, which meant that for diabetes, high blood pressure, and back pain, “ever” measures were used. For intermittent conditions like back pain, this “ever” definition will include both those who had the condition at age 42, and those who had suffered from back pain in the past but recovered. Analyses also do not account for cases arising between age 42 and 50-54 or for any recovery from these conditions during this period (e.g., weight loss from obesity). This may mean that our results underestimate of the impact on IN-HLT of more episodic, fluctuating conditions like mental ill-health and back pain, which interact dynamically with work across the life course (e.g., work stress impacts health and health in turn impacts the ability to work), and the unpredictable nature of which may make it more difficult to remain in work.

Finally, the comparison group in each of our models consisted of all individuals who did not report the given chronic condition and therefore includes both people without self-reported health problems, and those with health problems other than the condition of interest. Our results therefore likely underestimate the impact of chronic health conditions on IN-HLT relative to a “healthy” comparison group.

Secondly, economic activity was defined at the time of the interview, and was measured twice, at age 42 (lagged outcome) and at age 50-54 (outcome). Transitions into and out of IN-HLT occurring during this time window are therefore not captured in our study, and our approach does not identify how quickly transitions occurred. The impact of period effects on the measurement of economic activity at age 50-54 is also a challenge. In the 1958c, outcome measurement coincided with the start of the 2008 Great Recession, and in the 1970c, it occurred during the aftermath of the COVID-19 pandemic and the Cost-of-Living crisis. These period effects may have resulted in a higher proportion of people in both cohorts self-reporting IN-HLT as opposed to IN-OTH. Disability benefit claims can rise during times of financial crisis since rates of job loss are often higher among those with health problems, and disability benefits may be more generous and have lower conditionality than unemployment benefits (Pasini & Zantomio, 2013). Greater health-consciousness in the aftermath of the COVID-19 pandemic may also have influenced how members of the 1970c reported their economic activity. Nevertheless, IN-HLT is a policy-relevant outcome, and the ability to examine associations between chronic conditions and IN-HLT under a series of different economic and policy contexts can also be perceived as a strength.

Finally, like all longitudinal studies, the 1958c and 1970c experienced attrition over time. Cohort members experiencing poor health or who are socioeconomically disadvantaged are more likely to be non-respondents (Katsoulis et al., 2026; Mostafa et al., 2021), meaning that complete case analysis could underestimate the impact of ill-health on IN-HLT. To mitigate potential bias from of item- and unit non-response, we used MICE and IPWs. While poor health was associated with a higher risk of IN-HLT in complete case, unweighted analyses (Supplementary Material), AMEs tended to be larger when using MICE and IPW, particularly for the 1970c which has experienced greater loss to follow-up than the 1958c at comparable ages. Despite more than 7000 respondents in each cohort, analyses were underpowered for certain health outcomes due to low prevalence (e.g., diabetes) and for stratified analyses, where it was only possible to show a consistent direction in the association between health conditions and IN-HLT across population subgroups, but not meaningfully tease apart differences between cohorts or strata. Quantifying potential differences in the impact of chronic health conditions on IN-HLT between groups (e.g., by gender, race/ethnicity – participants in the 1958c and 1970c were almost exclusively white – or individual- and area-level deprivation) in addition to differences in the burden of ill-health experienced by these groups may help to better target support and interventions.

### 4.3 Implications

Chronic health conditions in early midlife are strongly associated with IN-HLT at age 50-54, more than a decade before State Pension Age. In the UK, labour market participation rates decline substantially even before State Pension Age (85% at age 50-54; 58% at 60-64; 23% at 65-69), and health reasons are one of the most commonly reported reasons for leaving the labour market among those not working at age 50-64, second only to retirement (DWP, 2024). Whilst for many individuals, IN-HLT and health-related benefits are a real necessity, reducing the rate and number of people who are IN-HLT at these older ages could be one way to mitigate the impact of population ageing on the size of the labour force. Improving the health of the working-age population is therefore not only valuable in its own right, but also economically advantageous (Crawshaw et al., 2024; Scott, 2021a, 2021b), with benefits beyond reducing IN-HLT.

Our findings suggest that it is important to both reduce the size of the population at risk of IN-HLT and weaken the impact of ill-health on IN-HLT if this is to be achieved. The proportion of adults in both cohorts experiencing chronic disease and mental ill-health at age 42 is already substantial, and preventative efforts, particularly primary prevention early in the lifecourse, are needed to reduce the prevalence of midlife ill-health in future generations. Early-life health is also important for a wide range of adult social and economic outcomes beyond IN-HLT, including household income and wealth, employment and occupation, educational attainment, and partnership status (e.g., Gondek et al., 2018; Goodman et al., 2011; Wu et al., 2025).

However, tackling the size of the population at risk in incoming cohorts of working-age individuals alone is unlikely to be sufficient to reduce rates of IN-HLT. Our study found that an individual with a chronic condition at age 42 born in 1970 was no less likely to be IN-HLT than someone born 12 years earlier after accounting for sociodemographic confounders, be this due to changes in the nature of health condition themselves, to changes in the reasons people give for being inactive, or to changes in factors modifying the impact of health on work participation.

While it is not known how the relationship between morbidity and IN-HLT has and will continue to change as younger cohorts enter midlife, increases in obesity and mental ill-health have already been observed in more recently born cohorts, and could result in a greater number of people IN-HLT unless the impact of chronic conditions on IN-HLT is weakened. This may involve interventions to improve the management of chronic conditions once they are diagnosed, including through occupational health and a better integration of care for co-occurring mental and physical health conditions (Henderson et al., 2024), and employment policies to lower barriers to remaining economically active, taking into consideration the importance of socioeconomic factors in employment outcomes.

Currently, only 1-in-6 people return to economic activity (including unemployment) within the first year of becoming IN-HLT, dropping to 1-in-20 for those who have been out of work for over a year (OBR, 2023). Strategies are therefore also needed to better support and reduce barriers for those who are IN-HLT to return to the labour market. A recent review of return-to-work policies in the UK has highlighted the paucity of robust evidence around what works to reduce economic inactivity among those experiencing long-term health conditions, with most existing interventions focusing heavily on the individual (e.g., sanctions, individualised support; Crawshaw et al., 2024; Haighton et al., 2025). Tackling the IN-HLT challenge is therefore a complex endeavour, and there is a need to consider approaches that also address the wider determinants of IN-HLT, including the contributions of low job quality and insecurity and local labour market conditions (Crawshaw et al., 2024).

## CRediT authorship contribution statement

**Laura Gimeno:** Writing – review & editing, Writing – original draft, Visualization, Project administration, Methodology, Investigation, Formal analysis, Data curation, Conceptualization. **Charis Bridger Staatz:** Writing – review & editing, Validation, Methodology, Data curation, Conceptualization. **Alice Goisis:** Writing – review & editing, Supervision, Conceptualization. **Jennifer B. Dowd:** Writing – review & editing, Supervision, Conceptualization. **George B. Ploubidis:** Writing – review & editing, Supervision, Methodology, Funding acquisition, Conceptualization.

## Ethics approval

Ethical approval for the 1958 National Child Development Study and the 1970 British Cohort Study was received from the National Health Service (NHS) Research Ethics Committees. All cohort members gave informed consent to participate in the study.

## Ethics approval

Ethical approval for the 1958 National Child Development Study and the 1970 British Cohort Study was received from the National Health Service (NHS) Research Ethics Committees. All cohort members gave informed consent to participate in the study.

## Funding

This research was funded by the Medical Research Council (grant number MR/N013867/1 to LG) and the European Research Council (grant number ERC-2021-CoG-101002587 to JBD). The Leverhulme Trust (grant number RC-2001-003) supports the Leverhulme Centre for Demographic Science at theUniversity of Oxford. The Economic and Social Research Council (grant numbers ES/M001660/1 and ES/W013142/1) supports the Centre for Longitudinal Studies at University College London. The funders had no role in the design of execution of this study, nor in the decision to publish.

## Declaration of competing interest

The authors declare that they have no known competing financial interests or personal relationships that could have appeared to influence the work reported in this paper.

## Data Availability

All data used in this study can be accessed by registered users free of charge through the UK Data Service (https://ukdataservice.ac.uk).
