# Supplementary material for: Chronic health conditions and health-related economic inactivity in midlife: Evidence from the 1958 and 1970 British birth cohorts
Source: SSM Popul Health. 2026 Jun 23;35:101940. doi: 10.1016/j.ssmph.2026.101940 (PMC13320332; doi:10.1016/j.ssmph.2026.101940)
Supplement: Multimedia component 1 [file mmc1.pdf]

Chronic health conditions and health-related economic inactivity in midlife: Evidence from the 1958 and 1970 British birth cohorts

Supplementary Material A

Table of Contents

Figure S1. Timeline of major disability legislation in the United Kingdom since 1970. 2

Table S1. Description of chronic health condition exposure measures used at age 42 in the 1958c and 1970c. 3

Text S1. Missing data strategy. 4

Table S2. Variables used in multiple imputation models. 5

Table S3. Number and percentage of observations among respondents at age 50-54 with missing information on exposures and covariates. 7

Table S4. Prevalence of chronic health conditions at age 42 and distribution of economic activity at ages 42 and 50-54. 8

Table S5. Distribution of economic activity at age 42 within categories of economic activity at age 50-54. 8

Table S6. Demographic and socioeconomic characteristics by cohort among respondents to age 50/51-54 sweeps. 9

Figure S2. Prevalence of chronic conditions at age 42 within economic activity groups at age 50-54 in the 1958c and 1970c. 10

Figure S3. Distribution of individuals with chronic conditions at age 42 by economic activity at age 50-54 in the 1958c and 1970c. 11

Figure S4. Relative risk ratios for chronic conditions at age 42 and IN-HLT at age 50-54 12

Figure S5. Average marginal effects for IN-HLT from fully adjusted models and sensitivity analyses..13

Figure S6. Average marginal effects for IN-HLT from fully adjusted, stratified models for longstanding illness and psychological distress. 14

Figure S1. Timeline of major disability legislation in the United Kingdom since 1970.

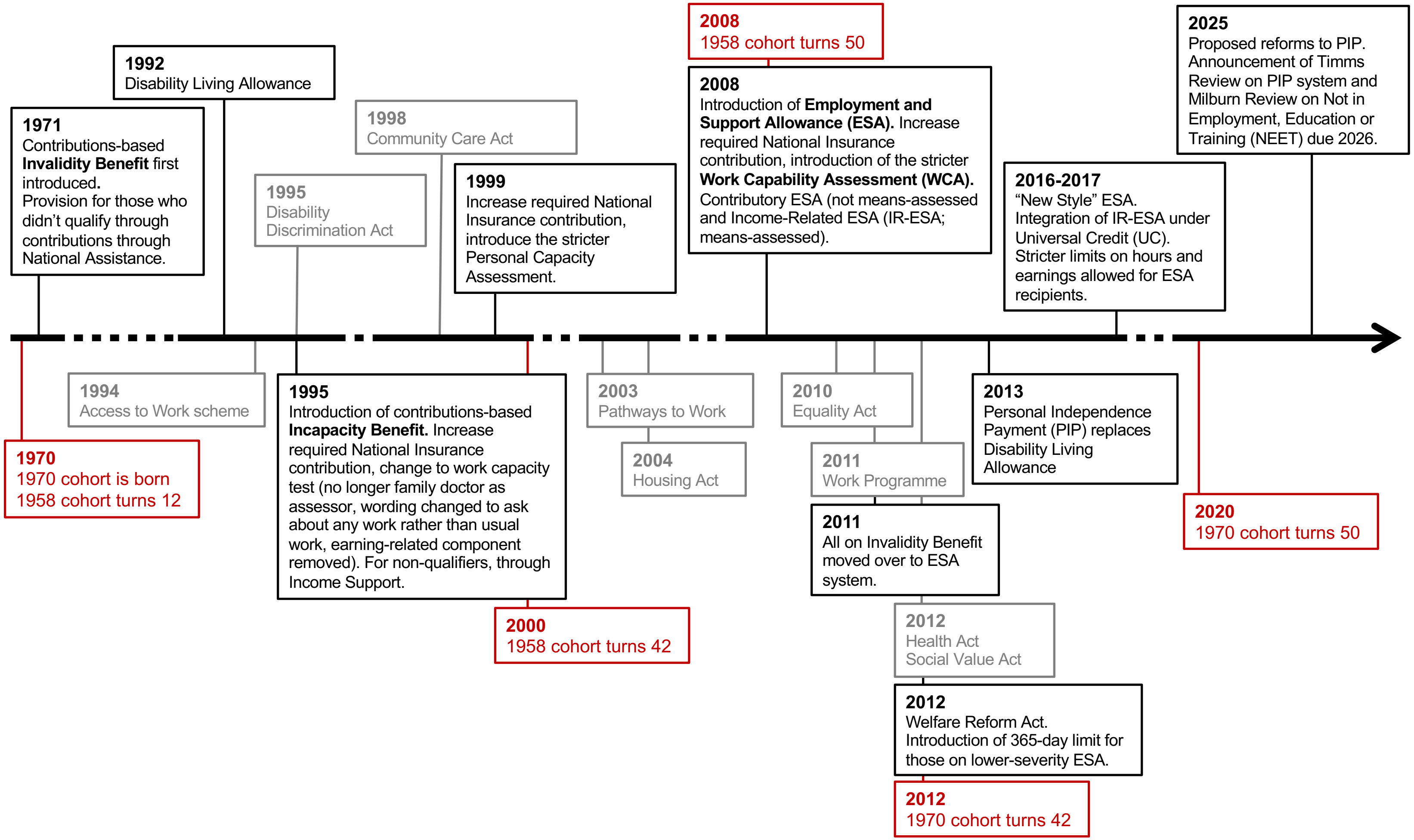

Note: Legislation and reforms related to the disability benefits system are shown in black. Legislation protecting the rights of those with disabilities, anti-discrimination laws and policies aiming to encourage participation of those with disability in employment are shown in grey. Major life events for the 1958 and 1970 cohorts are shown in red.

Table S1. Description of chronic health condition exposure measures used at age 42 in the 1958c and 1970c.

	**1958c**	**1970c**
Longstanding illness	Do you have any longstanding illness, disability or infirmity that has troubled you over a period of time or is likely to affect you over a period of time?	Do you have any physical or mental health conditions or illnesses lasting or expected to last 12 months or more?
Psychological distress[1]	9-Item Malaise Inventory, using a cut-off score of ≥4 (range 0-9).	9-Item Malaise Inventory, using a cut-off score of ≥4 (range 0-9).
Obesity[2]	Body mass index ≥30 kg/m^2 from harmonised self-reported height and weight.	Body mass index ≥30 kg/m^2 from harmonised self-reported height and weight.
Diabetes[3]	Harmonised indicator of lifetime diabetes prevalence, leveraging all parent reported and self-reported information from birth.	Harmonised indicator of lifetime diabetes prevalence, leveraging all parent reported and self-reported information from birth.
High blood pressure	Lifetime prevalence indicator from ever report of HIBP at age 42.	Lifetime prevalence indicator derived from ever report of HIBP at age 30, and reports of HIBP since last sweep at ages 34, 38 and 42.
Back pain	Lifetime prevalence indicator from ever report of back pain at age 42. Question asks about whether cohort member has experienced persistent back pain, sciatica, or slipped disc.	Lifetime prevalence indicator derived from ever report of back pain at age 30, and reports of back pain since last sweep at ages 34, 38 and 42. Questions ask about whether cohort member has experienced persistent back pain, sciatica, or slipped disc.

[1]This measure has been shown to exhibit scalar invariance across the two cohorts, suggesting that members of both cohorts interpret the items of the Malaise Inventory similarly (Ploubidis, Sullivan, Brown and Goodman, 2017. *Psychol Med;* 47(2): 291-303).

[2]Hardy, Johnson and Park, 2016. CLOSER Work Package 1: Harmonised height. Weight and BMI User Guide. London: CLOSER.

[3]Gimeno, Narayanan and Hardy, 2025. Harmonised indicators of self-reported diabetes in five British birth cohort studies. User Guide (Version 1). London: Centre for Longitudinal Studies.

Text S1. Missing data strategy.

Like all longitudinal studies, the 1958c and 1970c experience loss to follow-up, the likelihood of which varies by individual level characteristics, such as gender, health, and socioeconomic status. Over time, this results in the respondents being a non-representative subset of the cohort as it was initially sampled. We capitalised on the richness of the cohort data and the known properties of sample to restore sample representativeness at age 42 and 50-54, combining multiple imputation to handle item non-response, and inverse probability weights (IPW) to handle unit non-response. We used a similar approach for both the generation of descriptive statistics (chronic health condition prevalence, distribution by economic activity) at age 42, and to create the analysis datasets from which regression models were run.

To deal with item missingness amongst respondents at age 42 and/or at age 50-54, we imputed missing data using multivariate imputation by chained equations (MICE). Multiple imputation works under the assumption that data are Missing At Random (MAR), that is, that the observed data can explain systematic differences between the observed and missing values (White, Royston & Wood, 2011). However, this assumption is largely untestable. To make the MAR assumption more plausible, we included data on exposures, the outcome, all covariates, and a diverse set of auxiliary variables predictive of non-response or of the underlying missing values shown in Table S2 below (Mostafa et al, 2021; Katsoulis et al, 2024). Information on the amount of missing data is shown Table S3 below.

To account for unit non-response at age 42 and/or at age 50-54 (that is, people not responding to the survey sweep of interest), we derived IPWs for non-response. Weights were derived for the target population at each sweep, which is cohort members who were alive and living in the UK at the point of data collection (e.g., at age 50-54). Multiple imputation was used to create the data from which inverse probability weights were derived, to make sure that all cohort members could be included in the weight derivation process. We imputed data on all variables to be used in the regression models (one imputation model per sweep per study), creating 5 imputed datasets for each cohort. Each imputation model contained an indicator of sweep non-response (the outcome), and a range of predictors of non-response measured earlier in the lifecourse. These included sex at birth, parental social class, household overcrowding in childhood, cognitive ability and mental health in childhood/adolescence, measures of social capital/social participation (e.g., voting, membership in social/political organisations, social support, partnership status), socioeconomic status (e.g., educational attainment, whether in employment, income), health in adulthood (e.g., mental health, body mass index, self-rated general health, smoking status), and previous response to sweeps (i.e., number of previous sweeps cohort member had responded to).

Following imputation, we constructed logistic regression models (one per cohort) to predict the probability of responding to each sweep using imputed predictors (associated with non-response. We used the predicted probability of responding to the sweep based on observed characteristics to create inverse probability weights. We truncated weights to the value of 10 to prevent extreme weights exerting undue influence on our analyses. The weights were then rescaled to the respective sweeps, so that the sum of the weights was equal to the number of respondents at the sweep. The weighted distribution of sex at birth and parental socioeconomic status at birth in the age 42 and 50-54 analytical samples were similar to the distribution of these characteristics in the birth sweep.

Table S2. Variables used in multiple imputation models.

Variable	Description	Age measured		In model	
		1958c	1970c	1	2
Exposures					
LSI	Yes or no (binary)	42	42	X	X
PD	Yes or no (binary)	42	42	X	X
Obesity	Yes or no (binary)	42	42	X	X
Back pain	Ever or never (binary)	42	42	X	X
Diabetes	Ever or never (binary)	42	42	X	X
HIBP	Ever or never (binary)	42	42	X	X
Outcome/Lagged Outcome					
EconAct at 50-54	ACTFT, ACTPT, UNEMP, INHLT, INOTH (categorical)	50-51	51-54		X
EconAct at 42	ACTFT, ACTPT, UNEMP, INHLT, INOTH (categorical)	42	42	X	X
Socioeconomic controls or auxiliary variables					
Mother education	Age left full-time education (continuous)	0	0	X	X
Father education	Age left full-time education (continuous)	0	0	X	X
Father SEP	Skilled (RGSC I/II/IINM/IIM) or partly/unskilled (RGSC IV/V) (binary)	0	0	X	X
Childhood tenure	Parents owned home or other (binary)	7	5	X	X
Crowding	People in household divided by number of rooms in house excluding kitchen and bathrooms (ordinal/continuous)	7	5	X	X
Prior tenure	Owned home or other (binary)	33	34	X	X
Prior EconAct	ACTFT, ACTPT, UNEMP, INHLT, INOTH (categorical)	33	34	X	
Prior employment	Working or not working (binary)	33	34		X
Prior income	Quintiles of equivalised household income (ordinal)	33	34	X	X
Prior education	Degree (NVQ 4-5) or no degree (NVQ 0-3) (binary)	33	34	X	X
Prior occupation	Manual (RGSC IIIM-V) or non-manual (RGSC I-IIINM) (binary)	33	34	X	X
Tenure	Owned home or other (binary)	42	42		X
Income	Quintiles of equivalised household income (ordinal)	42	42		X
Education	Degree (NVQ 4-5) or no degree (NVQ 0-3) (binary)	42	42		X
Occupation	Manual (RGSC IIIM-V) or non-manual (RGSC I-IIINM) (binary)	42	42		X
Demographic controls or auxiliary variables					
Sex at birth	Male or female (binary)	0	0	X	X
Country of birth	England or other (binary)	0	0	X	X
Prior children	Number of dependent children (continuous)	33	34	X	X
Prior partnership	Married/cohabiting or other (binary)	33	34	X	X
Children	Whether has ≥1 dependent children (binary)	42	42		X
Partnership	Married/cohabiting or other (binary)	42	42		X
Region	North, West, East of England, or Wales/Scotland/Northern Ireland (categorical)	42	42		X

Table S2 continued next page

Table S2 continued

Variable	Description	Age measured		In model	
		1958c	1970c	1	2
Age	Age at interview in years at outcome measurement (continuous)	NA	51-54		X
Health controls or auxiliary variables					
Birthweight	Birthweight in grams (continuous)	0	0	X	X
Breastfed	Ever or never (binary)	0	0	X	X
Maternal smoking	Yes or no (binary)	0	0	X	X
Cognitive ability	Principal component 1 (continuous)	11	10	X	X
Chronic conditions during childhood	Whether had ≥1 of pathological heart condition, recurrent sore throat, recurrent abdominal pain, eczema, hay fever in the last 12 months (continuous)	11	10	X	X
Child MH	Rutter total score (continuous)	11	10	X	X
Child BMI	Body mass index in kg/m^2 (continuous)	11	10	X	X
Adolescent MH	Malaise Inventory total score (continuous)	16	16	X	X
Adolescent BMI	Body mass index in kg/m^2 (continuous)	16	16	X	X
Maternal MH	Malaise Inventory total score (continuous)	NA	16	X	X
Prior MH	Malaise Inventory total score (continuous)	33	34	X	X
Prior BMI	Body mass index in kg/m2 (continuous)	33	34	X	X
Prior LSI	Yes or no (binary)	33	34	X	X
Smoking	Current or non-smoker (binary)	33	34	X	X
SRH	Excellent-Good or Fair-Poor (binary)	33	34	X	X
Auxiliary variable: Previous response patterns					
Number of previous sweeps	Number of sweeps participated in prior to age 50/51-54 sweep (continuous)	0-33 OR 0-46	0-38 OR 0-46	X	X

Note: Model 1 = for descriptive analyses at age 42 (among respondents at age 42). Model 2 = for main analyses (among respondents at age 50-54). LSI = longstanding illness. PD = psychological distress. BMI = body mass index. HIBP = high blood pressure. EconAct = Economic Activity. ACTFT = Active full-time. ACT-PT = Active part-time. UNEMP = Unemployed. INHLT = Inactive due to health reasons. INOTH = Inactive due to other reasons. NVQ = National Vocational Qualification. RGSC = Registrar General Social Class. MH = Mental Health. SRH = Self Rated Health

Table S3. Number and percentage of observations among respondents at age 50-54 with missing information on exposures and covariates.

	**1958c (N = 9761) n (%)**	**1970c (N = 7336) n (%)**
**Exposures**		
Longstanding illness at age 42	697 (7.1)	598 (8.2)
Obesity at age 42	1565 (16.0)	1788 (24.4)
Diabetes at age 42	696 (7.1)	25 (0.3)
High blood pressure at age 42	703 (7.2)	236 (3.2)
Back pain at age 42	700 (7.2)	234 (3.2)
Psychological distress at age 42	768 (7.9)	1261 (17.2)
**Covariates**		
Sex at birth	0 (0)	0 (0)
Education at age 42	1 (<0.1)	573 (7.8)
Occupation at age 42	1952 (20.0)	1448 (19.7)
Household income quintile at age 42	2059 (21.1)	1821 (24.8)
Housing tenure at age 42	727 (7.5)	601 (8.2)
Partnership status at age 42	724 (7.4)	575 (7.8)
Cognitive ability at age 10/11	1327 (13.6)	2031 (27.7)
Region of residence at age 42	690 (7.1)	570 (7.8)
Children in household at age 42	704 (7.2)	570 (7.8)
Parental occupation at birth	932 (9.6)	568 (7.7)
Economic activity at age 42	695 (7.1)	582 (7.9)

Note: Based on imputed and weighted data. ACTFT = active full-time. ACTPT = active part-time. UNEMP = unemployed. INHLT = inactive due to health reasons. INOTH = inactive due to other reasons.

Table S4. Prevalence of chronic health conditions at age 42 and distribution of economic activity at ages 42 and 50-54.

	1958c<br>% (95% CI)	1970c<br>% (95% CI)
Chronic conditions at age 42		
Longstanding illness	30.8 (29.8-31.8)	31.1 (29.9-32.3)
Obesity	16.8 (15.9-17.7)	23.9 (22.6-25.2)
Diabetes	2.6 (2.3-3.0)	3.3 (2.9-3.8)
High blood pressure	11.9 (11.2-12.6)	13.5 (12.7-14.3)
Back pain	23.1 (22.2-24.1)	31.0 (29.9-32.1)
Psychological distress	14.2 (13.4-15.0)	21.4 (20.1-22.6)
Economic activity at age 42		
ACTFT	64.8 (63.7–65.8)	63.3 (62.1–64.6)
ACTPT	17.6 (16.9–18.4)	18.4 (17.4–19.3)
UNEMP	2.7 (2.3–3.1)	3.6 (3.0–4.2)
INHLT	5.8 (5.2–6.4)	5.0 (4.4–5.6)
INOTH	9.1 (8.5–9.8)	9.7 (8.9–10.5)
Economic activity at age 50-54		
ACTFT	64.0 (62.6–65.4)	64.6 (62.8–66.4)
ACTPT	15.9 (15.0–17.0)	16.8 (15.6–18.1)
UNEMP	3.3 (2.7–3.9)	2.0 (1.4–2.7)
INHLT	7.8 (6.8–8.7)	8.8 (7.4–10.2)
INOTH	8.9 (7.9–9.9)	7.7 (6.8–8.7)

Note: Based on imputed and weighted data. ACTFT = active full-time. ACTPT = active part-time. UNEMP = unemployed. INHLT = inactive due to health reasons. INOTH = inactive due to other reasons.

Table S5. Distribution of economic activity at age 42 within categories of economic activity at age 50-54.

		**Activity @50-54**				
		ACTFT<br>%	ACTPT<br>%	UNEMP<br>%	INHLT<br>%	INOTH<br>%
**Activity @42**	***1958c***					
	ACTFT	85.9	21.7	54.3	29.4	22.8
	ACTPT	8.0	59.8	10.9	9.8	17.8
	UNEMP	2.0	1.3	26.9	3.3	0.9
	INHLT	1.2	1.5	1.3	51.7	1.2
	INOTH	2.9	15.7	6.5	5.8	57.3
	1970c					
	ACTFT	84.3	22.5	28.9	22.3	31.8
	ACTPT	10.0	60.2	16.9	14.3	14.5
	UNEMP	2.1	2.1	45.2	6.9	5.1
	INHLT	0.4	0.6	1.9	50.2	0.8
	INOTH	3.2	14.5	7.0	6.2	47.8

Note: ACTFT = active full-time. ACTPT = active part-time. UNEMP = unemployed. INHLT = inactive due to health reasons. INOTH = inactive due to other reasons. Based on imputed and weighted data.

Table S6. Demographic and socioeconomic characteristics by cohort among respondents to age 50/51-54 sweeps.

	**1958c (n = 9761) % (95% CI)**	**1970c (n = 7337) % (95% CI)**
Male sex at birth	50.7 (49.2-52.1)	49.6 (47.9-51.3)
Degree-level qualifications at 42	28.6 (27.5-29.7)	36.9 (35.2-38.7)
Manual occupation at 42	41.1 (36.0-46.2)	33.6 (30.8-36.4)
Owns or is buying own home at 42	76.6 (74.8-78.5)	67.1 (64.8-69.4)
Married or cohabiting at 42	78.1 (76.1-80.1)	59.0 (56.8-61.3)
Parent in partly/unskilled job at age 10/11	21.9 (99.9-23.9)	22.5 (20.7-24.3)
Has children in household at age 42	73.8 (71.9-75.7)	71.8 (70.0-73.8)
Equivalised household income at 42		
1 (lowest quintile)	21.8 (20.2-23.3)	24.4 (22.2-26.7)
2	20.4 (18.9-21.8)	20.5 (18.6-22.4)
3	19.4 (17.9-20.9)	19.5 (17.9-21.1)
4	19.6 (18.2-21.1)	18.3 (16.8-19.7)
5 (highest quintile)	18.8 (17.4-20.3)	17.3 (16.0-18.9)
Region of residence[1] at age 42		
North of England	24.9 (23.1-26.7)	26.9 (24.9-28.9)
West of England	19.3 (17.4-21.2)	19.1 (17.4-20.9)
East of England	42.3 (41.1-44.8)	42.0 (40.0-44.1)
Wales/Scotland/Northern Ireland	12.8 (11.8-13.8)	11.9 (10.6-13.2)

Note: Means and percentage shown in the table are based on imputed and weighted data. [1]North of England = North East, North West, Yorkshire & The Humber. West of England = West Midlands, South West. East of England = East Midlands, East Anglia, South East and London.

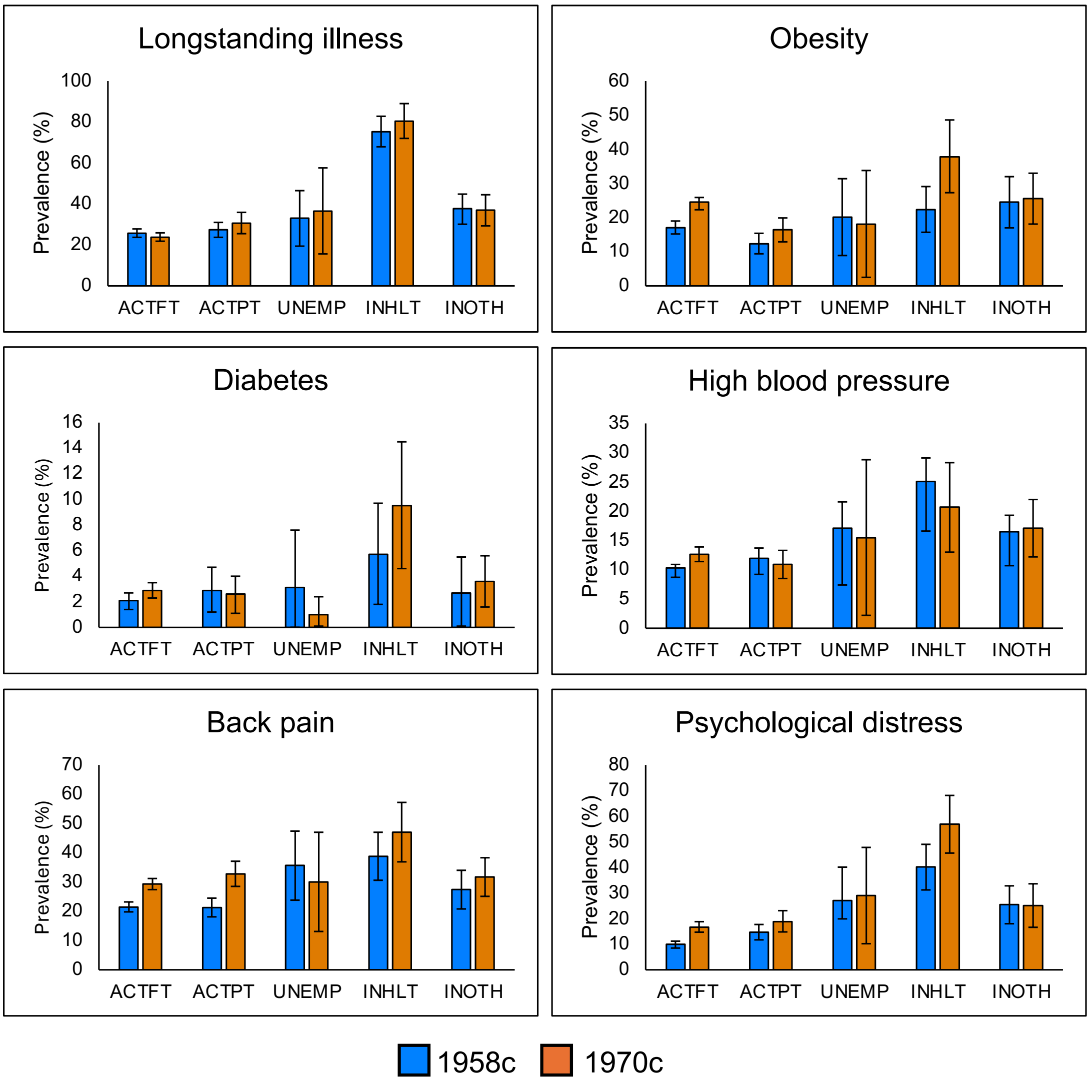

Figure S2. Prevalence of chronic conditions at age 42 within economic activity groups at age 50-54 in the 1958c and 1970c.

Note: ACTFT = active full-time. ACTPT = active part-time. UNEMP = unemployed. INHLT = inactive due to health reasons. INOTH = inactive due to other reasons. Based on multiply imputed and weighted data.

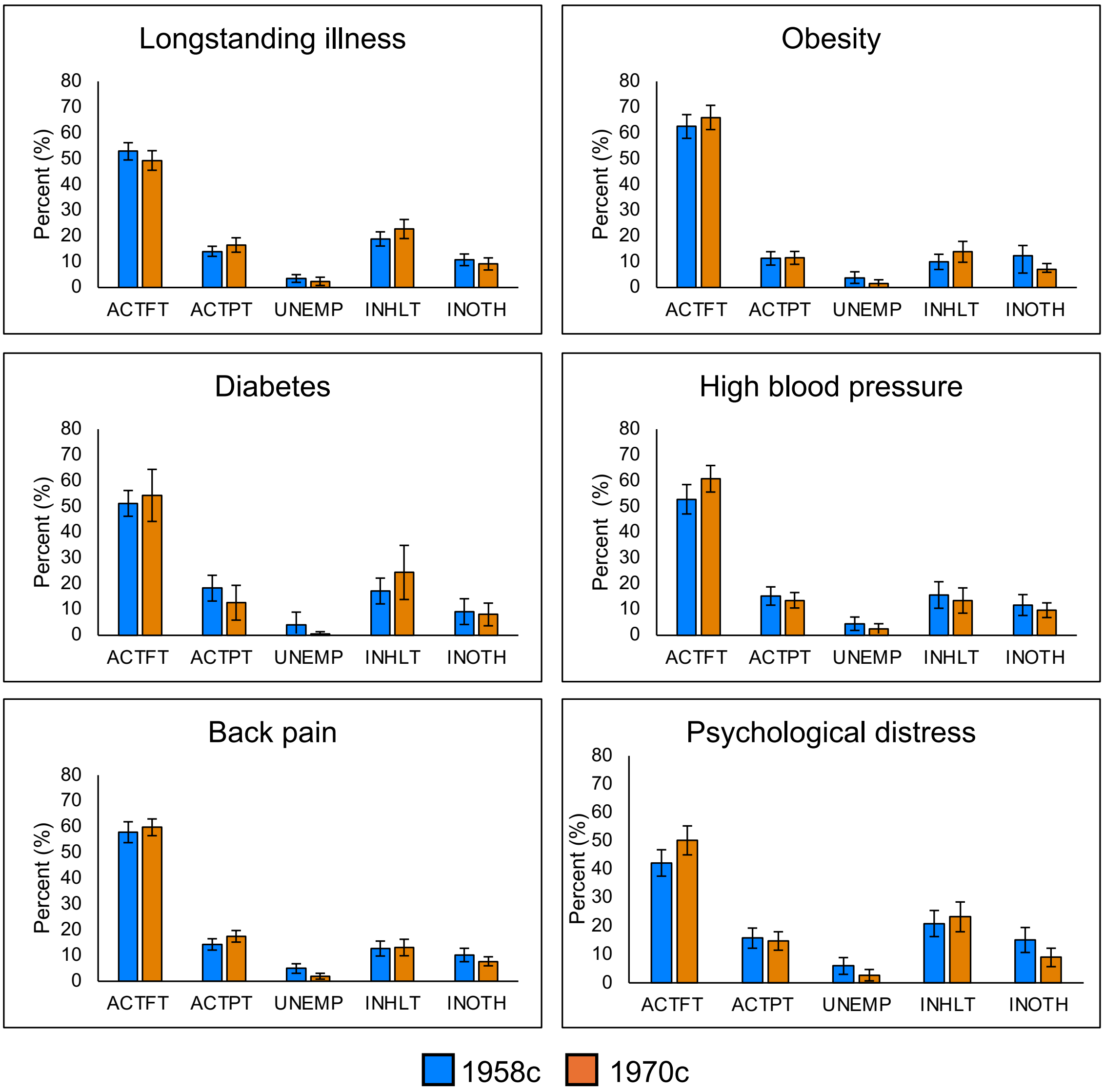

Figure S3. Distribution of individuals with chronic conditions at age 42 by economic activity at age 50-54 in the 1958c and 1970c.

Note: ACTFT = active full-time. ACTPT = active part-time. UNEMP = unemployed. INHLT = inactive due to health reasons. INOTH = inactive due to other reasons. Based on multiply imputed and weighted data.

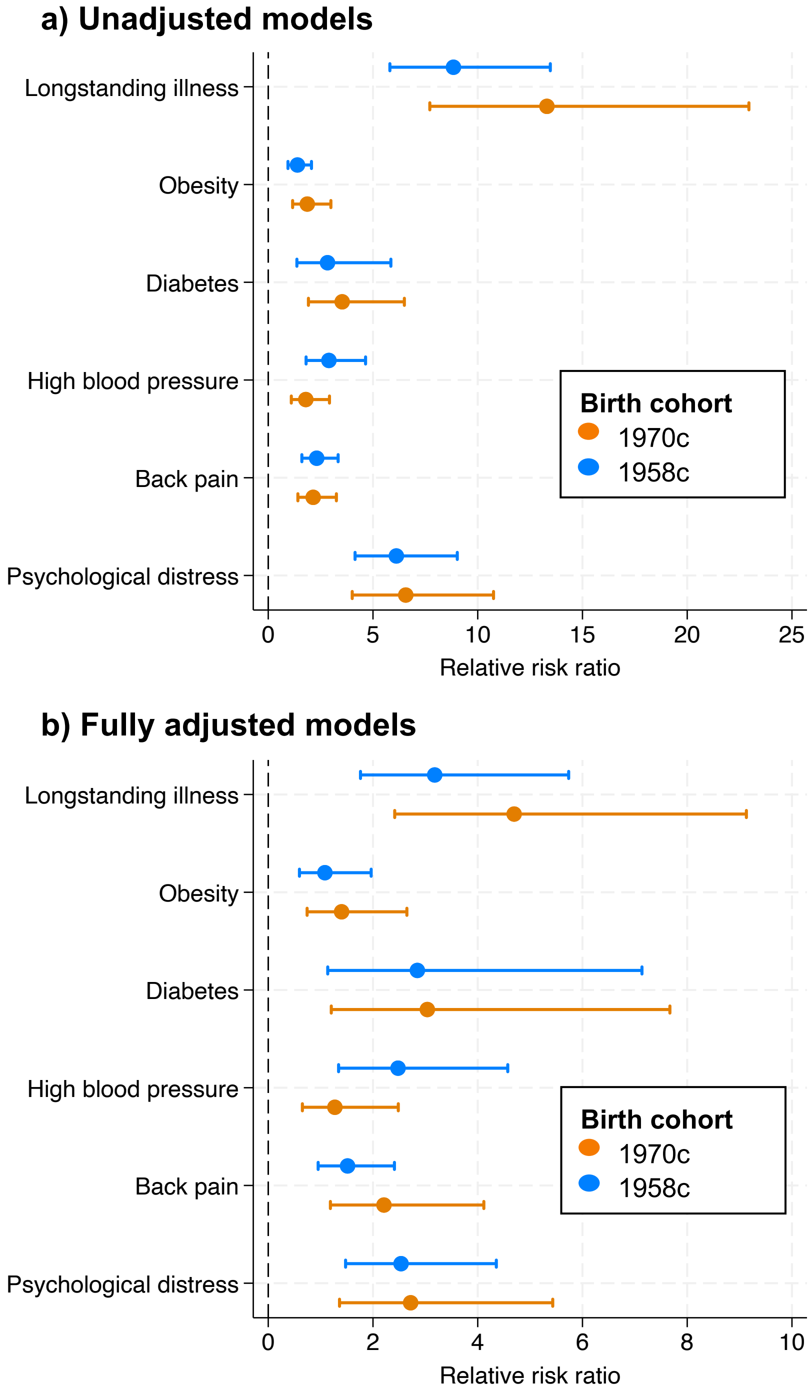

Figure S4. Relative risk ratios for chronic conditions at age 42 and IN-HLT at age 50-54

Note: Baseline group for the outcome is those active full-time (ACT-FT). Unadjusted models include on the exposure and outcome (economic activity at age 50-54). Fully adjusted models control for sex and parental social class at birth, cognitive ability at age 10/11, and education, occupation, household income, housing tenure, partnership status, region of residence, and whether the cohort member had any children in the household at age 42. In models for the 1970c, models are also adjusted for age at outcome measurement. Based on imputed and weighted data.

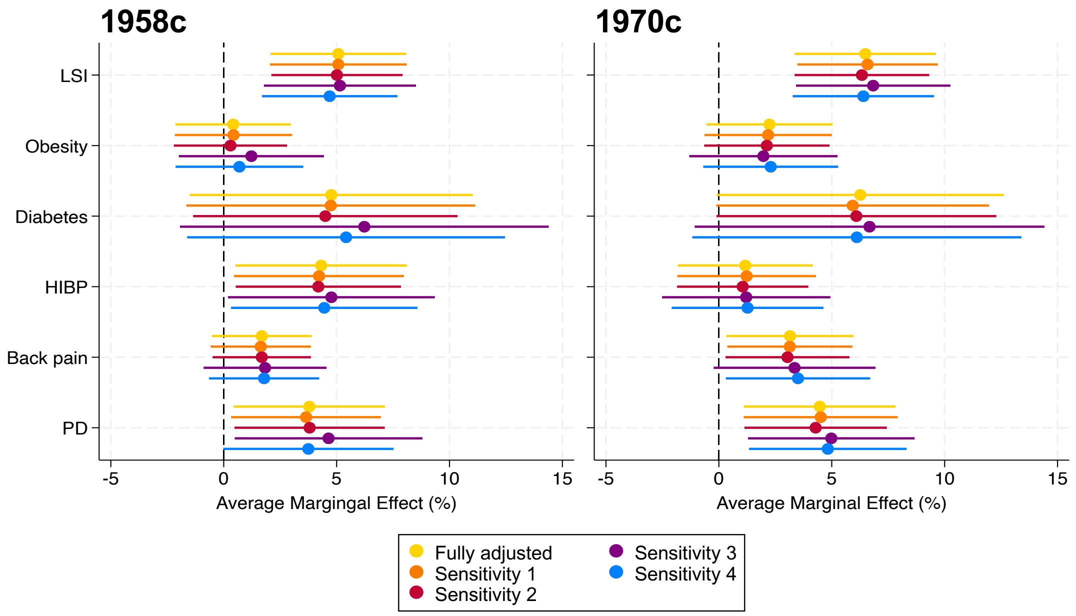

Figure S5. Average marginal effects for IN-HLT from fully adjusted models and sensitivity analyses

Note: LSI = longstanding illness. HIBP = high blood pressure. PD = psychological distress. Sensitivity 1 regroups ACT-FT, ACT-PT and UNEMP into a single category. Sensitivity 2 adjusts for additional potential confounders from early life. Sensitivity 3 restricts models to those ACT-FT, ACT-PT or UNEMP at age 42. Sensitivity 4 removes those IN-HLT at age 42 from the analytical sample. Based on imputed and weighted data.

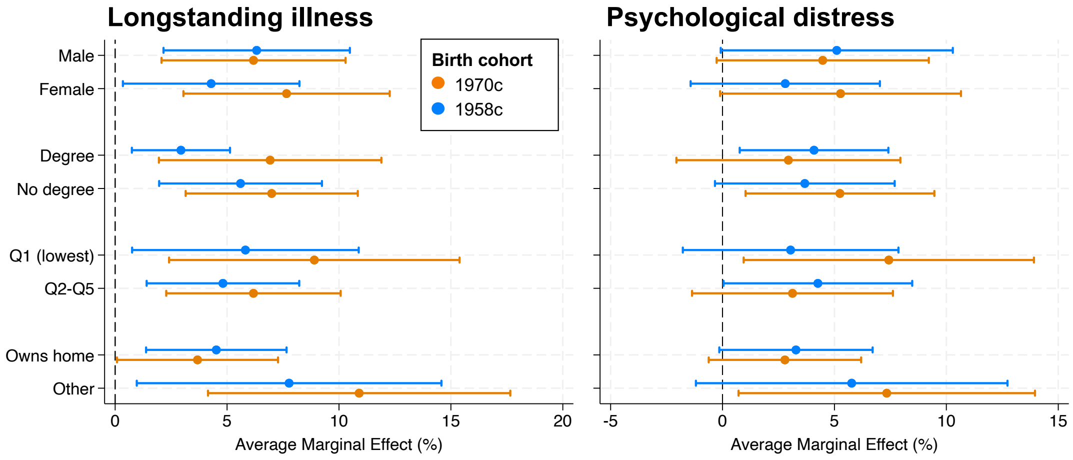

Figure S6. Average marginal effects for IN-HLT from fully adjusted, stratified models for longstanding illness and psychological distress.

Note: Models are adjusted for sex and parental social class at birth, cognitive ability at age 10/11, and education, occupation, household income, housing tenure, partnership status, region of residence, and whether the cohort member had any children in the household at age 42. Models for the 1970c also adjust for age at outcome measurement. The outcome variable is categorical with three levels: active, IN-HLT, and IN-OTH (with ACT-FT). Based on imputed and weighted data.
